# The Efficiency of an Integrated Program Using Falconry to Deter Gulls from Landfills

**DOI:** 10.3390/ani5020214

**Published:** 2015-04-10

**Authors:** Ericka Thiériot, Martin Patenaude-Monette, Pierre Molina, Jean-François Giroux

**Affiliations:** 1 Groupe de Recherche en Écologie Comportementale et Animale, Département des sciences biologiques, Université du Québec à Montréal, P.O. Box 8888 Station Centre-ville, Montréal, QC H3C 3P8, Canada; E-Mails: erickathieriot@hotmail.com (E.T.); mpatenaudemonette@gmail.com (M.P.-M.); 2 Falcon Environmental Services, P.O. Box 1018, St-Lazare, QC J7T 2Z7, Canada; E-Mail: pierre@faucon.biz

**Keywords:** anthropogenic food, bird control, culling, falconry, gull, landfill, *Larus delawarensis*, pest management, scaring

## Abstract

**Simple Summary:**

We evaluated the long-term effect of an intensive integrated program based on falconry to deter gulls, mostly ring-billed gulls (*Larus delawarensis*), from a landfill. Gulls were counted at different periods each day, and the annual sum of the maximum count at any observation period each day declined from over 1.1 million to only 20,300 during the 20 years of the study. This could not be explained by a decline in the local breeding population that remained relatively large throughout this period as determined in a concomitant study. The effectiveness of the falconry program was also confirmed by tracking individual birds fitted with GPS data loggers. The tagged gulls stopped less often and spent less time at the landfill with falconry than at another one where a selective culling program was conducted. We conclude that the use of an integrated program using falconry, which we consider more socially acceptable than culling, can be effective in deterring gulls from landfills.

**Abstract:**

Gulls are commonly attracted to landfills, and managers are often required to implement cost-effective and socially accepted deterrence programs. Our objective was to evaluate the effectiveness of an intensive program that integrated the use of trained birds of prey, pyrotechnics, and playback of gull distress calls at a landfill located close to a large ring-billed gull (*Larus delawarensis*) colony near Montreal, Quebec, Canada. We used long-term survey data on bird use of the landfill, conducted behavioral observations of gulls during one season and tracked birds fitted with GPS data loggers. We also carried out observations at another landfill located farther from the colony, where less refuse was brought and where a limited culling program was conducted. The integrated program based on falconry resulted in a 98% decrease in the annual total number of gulls counted each day between 1995 and 2014. A separate study indicated that the local breeding population of ring-billed gulls increased and then declined during this period but remained relatively large. In 2010, there was an average (±SE) of 59 ± 15 gulls/day using the site with falconry and only 0.4% ± 0.2% of these birds were feeding. At the other site, there was an average of 347 ± 55 gulls/day and 13% ± 3% were feeding. Twenty-two gulls tracked from the colony made 41 trips towards the landfills: twenty-five percent of the trips that passed by the site with falconry resulted in a stopover that lasted 22 ± 7 min compared to 85% at the other landfill lasting 63 ± 15 min. We concluded that the integrated program using falconry, which we consider more socially acceptable than selective culling, was effective in reducing the number of gulls at the landfill.

## 1. Introduction

Landfills represent predictable and nearly limitless food sources for scavenging birds [1,2]. Several species of gulls have adapted to anthropogenic environments and have learned to take advantage of waste disposal [3,4]. However, large numbers of gulls at landfills can be a nuisance for nearby residents when gulls loaf on their properties or defecate while in flight over their houses. Bacterial contamination of water has been associated with the presence of gulls [5]. Flocks of gulls can also be a risk to aircrafts when landfills are located near airports [6]. Collisions generally entail considerable economic losses and may result in human deaths [7,8,9]. Large numbers of gulls flying around heavy machinery at landfill sites can reduce the operators’ visibility with an increased risk of accidents. The constant noisy calling of gulls is an important nuisance for these staff, and along with the heavy fecal deposition on machinery, causes a significant health and safety issue. Finally, gulls can improve their reproductive success by feeding at landfills, which may contribute to population growth [10,11]. There is therefore a need to develop efficient and socially acceptable methods to deter gulls from using landfill sites.

Landfill management is the first step to minimize the number of birds. For instance, reducing the active tipping area surface, that is the area where waste is dumped from the trucks, and regular covering of the refuse with inedible materials should limit access to garbage and make the site less attractive to gulls. Avoiding water accumulation in shallow depressions and sowing tall grasses in inactive zones should prevent gulls from using these sites for preening and loafing [12]. Overhead wires have been used to exclude gulls from wastewater treatment plants and roofs [13,14]. However, the mobile machinery and the regular displacement of tipping areas preclude the use of overhead wires at large landfills. Active scaring programs that rely upon escape behavior [15] thus remain an essential method to deter gulls from using landfills, especially near the tipping areas. Short-term effects of various deterrence programs have been established in many studies but their long-term effects have been rarely documented [16,17,18,19].

Our objective was to assess the effectiveness of a comprehensive program based on falconry to deter gulls from using a landfill located near a large breeding colony of ring-billed gulls (*Larus delawarensis*). Although the ring-billed gull was the main species of concern, some herring (*L. argentatus*) and great black-backed gulls (*L. marinus*) were also subject to deterrence, especially in fall. Because deterrence is more efficient when several techniques are combined [16,17,18], the program integrated the use of trained birds of prey, pyrotechnics and playback of gull distress calls. We based our evaluation on both long-term survey data and detailed behavioral observations depicting landfill use by gulls. There was no other nearby comparable landfill that could serve as a control site. Nevertheless, we conducted detailed observations at another landfill located farther from the colony, where less waste material was brought and where a limited deterrence program based on selective culling and pyrotechnics took place [19]. We postulated that the integrated program based on falconry could be considered effective if the number of gulls using the landfill declined over time and if the use by gulls was less than at the other less attractive landfill. We also tracked individual gulls from the colony to both landfills to study their foraging behavior in relation to the sites’ attractiveness and deterrence programs.

## 2. Methods

### 2.1. Study Area

We conducted the study at the Terrebonne and Ste-Sophie landfills in the vicinity of Montreal, Quebec, Canada. These sites were respectively located 8 and 37 km from a ring-billed gull colony located on Deslauriers Island in the St-Lawrence River. Triennial surveys in this colony showed that the size of the breeding population increased from 97,600 birds in 1994 to 102,000 in 2006 and then declined to 87,800 in 2012 [20]. Adults start to arrive on the colony in late March. They establish their territory, lay their eggs and start to incubate in April. The brood rearing lasts from mid-May to late June, which corresponds to the period with the highest food demand as young are fed by their parents on the colony. Most adults and fledged juveniles have left the colony by early July [19].

The area surrounding the Terrebonne site included suburban settlings and agricultural lands while the Ste-Sophie site was entirely surrounded by agricultural lands. An average of 854,500 ± 46,300 (SE) tons of refuse were brought annually to the Terrebonne site between 1995 and 2003 and this increased to an average of 1,277,800 ± 12,000 tons between 2004 and 2014. At Ste-Sophie, 731,000 and 985,000 tons were brought during the study in 2009 and 2010, respectively. At both landfills, refuse was dumped and compacted in an active tipping area where it was rapidly covered with earth or inedible material. The other portions of the landfills were covered with grass, clay, or sand. Burying operations took place daily except for Sundays.

### 2.2. Control Programs

At the Terrebonne landfill, trained wildlife-control officers (WCO) strictly dedicated to gull control maintained an integrated deterrence program between 1995 and 2014. This involved flying captive bred gyrfalcons (*Falco rusticolus*), saker falcons (*F. cherrug*), and Harris’s hawks (*Parabuteo unicinctus*). A hawk can fly up to 25 min per day (5 min per flight) and typically three hawks were active on the site totaling approximately 75 min of flight in a typical day. In comparison, a falcon flies up to 20 min per day (10 min per flight) and two birds were used for a total of 40 min. WCO flew falcons above the site with a lure whereas they trained the hawks to catch gulls that came near the ground and to fly among groups of gulls. To achieve the greatest impact, recorded distress calls were broadcasted to attract gulls near the WCO before launching the birds of prey. As an integral component of the deterrence program, WCO also fired pyrotechnics (Screamers, Margo Supplies Ltd., Alberta, Canada). The number of shots depended of the abundance of gulls at the landfill, which varied with their breeding cycle. In 2010, the number of pyrotechnics shot per hour averaged 2.0 ± 0.4 during the nesting period, 6.7 ± 0.8 during the brood rearing period and 1.6 ± 0.3 after the breeding season. Deterrence took place during 8 to 12 h per day until 2004 but was extended from dawn to dusk starting in 2005. The mean number of hours of deterrence per year averaged 1227 between 1995 and 2004 and increased to 4511 thereafter. Starting in 2006, two WCO were involved during week-days and one on weekends but the number could reach up to five on week-days when gull abundance increased. The period with deterrence activities slightly varied among years but generally occurred from March to December.

At the Ste-Sophie landfill, the deterrence program was only performed on week-days between 0700 and 1500 by a site employee unspecialized in wildlife control. Selective culling involved shooting of a maximum of 21 gulls per week in 2009 and 35 in 2010 using a 12-gauge gun with 3-inch BB steel shots. In 2010, an average of 19 steel rounds were used per day. Culling occurred from 1 April to 30 November and was also combined with the use of the same pyrotechnics as those used at Terrebonne. The number of shots per hour averaged 1.0 ± 0.4, 2.4 ± 0.5, and 1.4 ± 0.7 during the nesting, brood rearing and post-breeding stages, respectively. In 2010, an experiment was conducted between April and August to compare the effectiveness of culling and the use of rubber shots [19]. Trials lasted seven days, with five replicates for culling and four for rubber shots with a three-day non-deterrence period between each trial. After this experiment, the selective culling program was resumed until the end of November.

### 2.3. Gull Surveys

At Terrebonne, we conducted daily surveys from 1995 to 2014 in the morning, mid-day and afternoon when deterrence took place. We counted all gulls observed within a 200-m radius of the active tipping area. We used the maximum number of gulls counted during any one observation period each day and summed these maxima across each year to obtain the number of gull-days per year. For days with missing data, we took the mean between the previous and next counts. To standardize the sampling effort, we restricted the analysis to the period between 1 April and 13 December when counts were available for all years.

During the breeding season of 2010, we conducted detailed observations at both landfills. We counted birds every 30 min during 5-h observation bouts alternating between three daily periods: morning (0500–1000), mid-day (1000–1500) and afternoon (1500–2000). For each count, we determined the number of birds on the entire site and at the tipping area. We also determined the proportion of each gull species and the proportion of birds feeding that was considered as the time spent feeding [21]. At each site, we tallied 29 days of observation distributed among three stages based on the breeding chronology of ring-billed gulls at Deslauriers Island in 2010: (1) the nesting stage lasted from 5 April to 14 May and coincided with nest establishment, egg laying and incubation; (2) the rearing stage took place between 15 May and 25 June and corresponded to the period when adults have to feed juveniles; and (3) the post-rearing stage from 26 June to 7 August concurred with the departure of gulls from the colony when juveniles can feed by themselves. At Ste-Sophie, we excluded days with the rubber shot trials and the associated control days, as this treatment was ineffective [19].

### 2.4. Telemetry

In 2009 and 2010, we used GPS-tracking devices to determine how ring-billed gulls breeding on Deslauriers Island reacted when flying near the studied landfills during their foraging trips. We captured and recaptured the gulls with nest traps or dip nets and fitted them with 10–15 g GiPSy-2 data loggers (Technosmart, Italy) that represented 2.8% ± 0.5% of the body mass of the birds (485 ± 49 g). We attached the loggers on the two median rectrices with white TESA tape (no. 4651) and programmed them to acquire locations (±5 m) at 4-min intervals for 2–3 days [22]. Based on the maximum flying speed of black-headed gulls (*L. ridibundus*, 14.7 m/s) and lesser black-backed gulls (*L. fuscus*, 15.5 m/s) [23], which are respectively slightly smaller and larger than ring-billed gulls, we calculated that a gull could cover 3–4 km during 4 min, and this exceeded the area occupied by the landfills. We thus considered that a single location above a landfill site represented a bird that passed through without stopping, whereas two or more locations represented a stopover that lasted 8 min or more depending on the number of locations. At Ste-Sophie, we only considered days with culling or weekends.

### 2.5. Statistical Analyses

We tested the difference in the number of gull-days between the first and last year of the surveys at the Terrebonne landfill with a Wilcoxon signed-rank test. We evaluated bird use of the two landfills in 2010 based on the mean daily numbers of birds computed from the repeated surveys. We assumed that each day was independent because the GPS-tracked birds did not return the following day to the visited landfill in 55% of the cases (*n* = 20 trips), indicating some turnover. We calculated the proportion of gulls feeding by dividing the total number of birds observed feeding by the total number present each day and converted this into a percentage. The relative importance of ring-billed gulls compared to the other two species was computed in the same way. We analyzed the number of gulls present at the whole site and at the active tipping area as well as the percentage of gulls feeding with ANOVAs including site, biological stage and their interactions as independent variables. We tested the effect of the daily period (morning, midday, afternoon) on the number of gulls and the percentage of birds feeding using one-way ANOVAs. We also compared the percentage of ring-billed gulls at both sites using ANOVA. We transformed count data with square roots to respect normality and applied angular transformations to percentages. We used *t*-tests and Tukey’s honestly significant difference tests to check for differences between each pair of means. We analyzed the proportion of foraging trips that resulted in a stopover at each landfill with a χ^2^ test while we compared the duration of the stopovers with a *t*-test. We established the statistical level of significance at 0.05 and presented means ± 1 SE.

## 3. Results

### 3.1. Gull Use of Landfills

**Figure 1:**
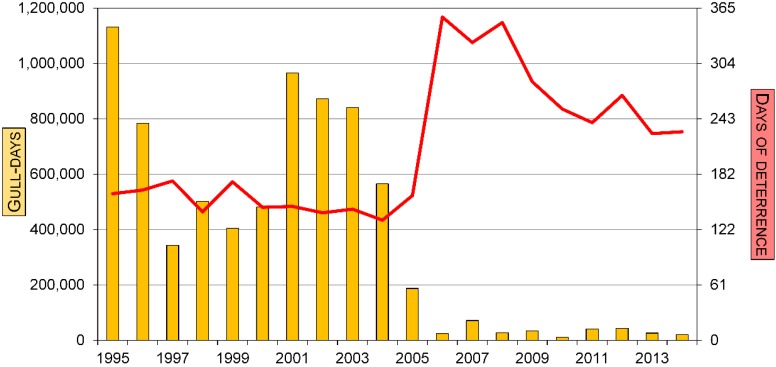
Use of the Terrebonne landfill by gulls and the number of days with deterrence activities, 1995–2014. The number of gull-days represents the sum of the maximum number of gulls observed during any one observation period each day between 1 April and 13 December each year.

We observed a 98% decline in gull use at the Terrebonne site between 1995 and 2014 (*W* = 33153, *p* < 0.001; Figure 1). The decline was notably accentuated starting in 2005–2006 when deterrence was intensified with more days of activities, more WCO and the extension of the deterrence hours from dawn to dusk.

In 2010, ring-billed gulls represented 99% ± 1% and 92% ± 2% of the gulls observed between 1 April and 31 August at Terrebonne and Ste-Sophie, respectively (*F*_1,184_ = 54.78, *p* < 0.001). Both herring and great black-backed gulls became increasingly more abundant as fall progressed (E. Thiériot and P. Molina, unpublished data).

**Figure 2:**
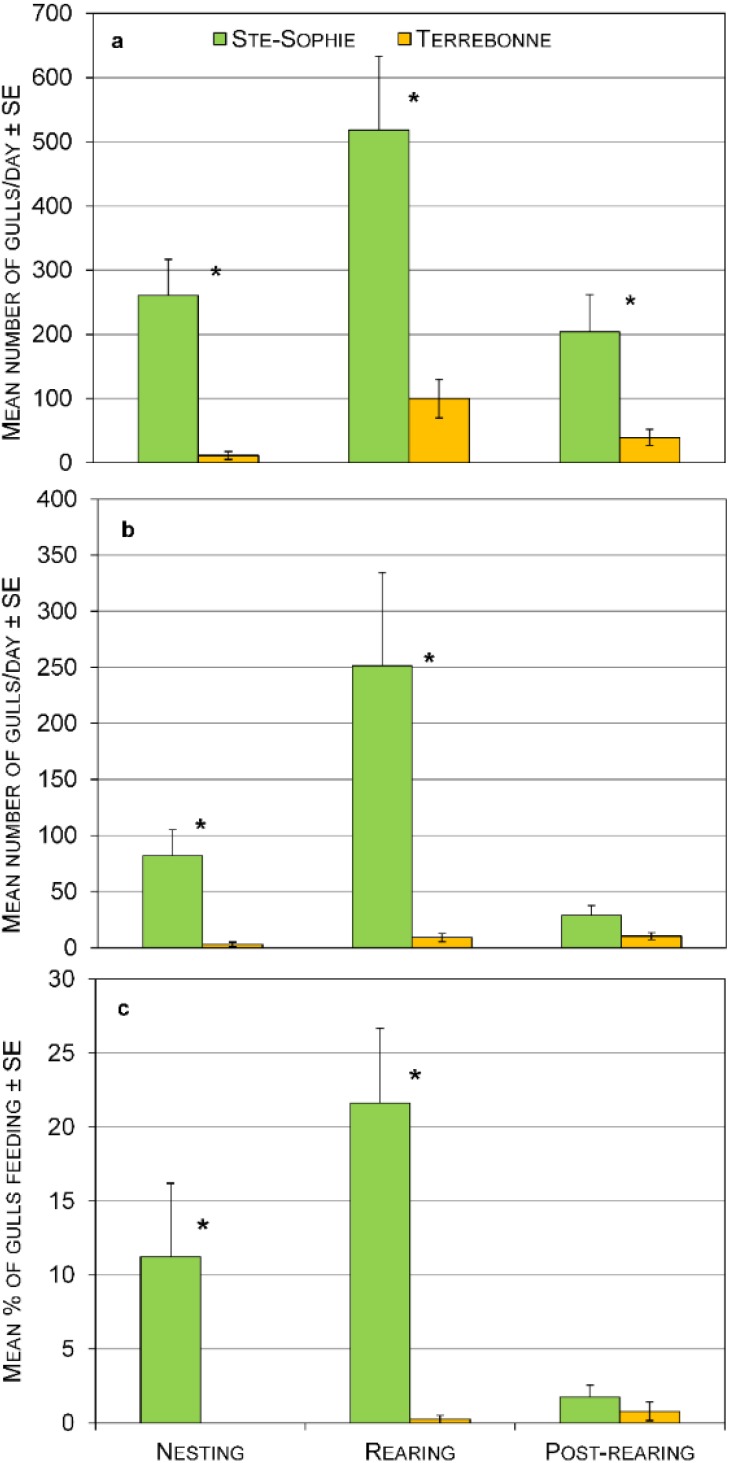
Use of the Terrebonne and Ste-Sophie landfills during the nesting, rearing, and post-rearing stages of ring-billed gulls breeding on Deslauriers Island, QC, 2010: (**a**) Mean ± SE number of gulls/day at each site; (**b**) Mean ± SE number of gulls/day at the tipping area at each site; (**c**) Mean ± SE percent of gulls feeding at each site. Asterisks represent significant differences between sites (*p* < 0.05).

We recorded fewer gulls using the Terrebonne than the Ste-Sophie landfill during all three stages (*F*_1,54_ = 50.43, *p* < 0.001; Figure 2a). The mean number of gulls was greater during the rearing stage at both sites (*F*_2,54_ = 6.06, *p* = 0.004). There was no interaction between these factors, indicating that the relative use of each site was similar throughout the breeding season. At Ste-Sophie, the mean number of gulls also depended on the daily period (*F*_2,26_ = 8.61, *p* = 0.001). More gulls were present after 1500 (582 ± 108 gulls/day) when culling activity had ceased compared to morning (181 ± 36 gulls/day) or mid-day periods (225 ± 44 gulls/day). There was no difference between daily periods at the Terrebonne site (*F*_2,26_ = 1.45, *p* = 0.253).

Very few gulls used the active tipping area at Terrebonne compared to Ste-Sophie and the difference was significant for the first two stages (*F*_1,52_ = 29.87, *p* < 0.001; Figure 2b). The mean number of gulls also varied according to biological stages (*F*_2,52_ = 5.31, *p* = 0.008) but there was a significant interaction between sites and stages (*F*_2,52_ = 5.45, *p* = 0.007). The biological stage had no effect at Terrebonne under the falconry program whereas there were more gulls at the tipping area at Ste-Sophie during the rearing stage. The mean number of gulls using the active tipping area also depended on the daily period at Ste-Sophie (*F*_2,26_ = 10.71, *p* = 0.000). More gulls were present after 1500 (283 ± 78 gulls/day) compared to morning (48 ± 13 gulls/day) or mid-day periods (41 ± 12 gulls/day). At Terrebonne, there was also a difference (*F*_2,26_ = 5.16, *P* = 0.01) between morning (3 ± 2 gulls/day) and mid-day periods (16 ± 4 gulls/day) but there was no difference between morning and evening (6 ± 2 gulls/day) or between mid-day and evening.

The percentage of gulls feeding on refuse was higher at Ste-Sophie than at Terrebonne but was globally low (*F*_1,52_ = 34.81, *p* < 0.001; Figure 2c). It varied according to biological stages (*F*_2,52_ = 5.75, *p* = 0.006) in interaction with sites (*F*_2,52_ = 6.91, *p* = 0.002). The biological stage had no effect at Terrebonne where <1% of the birds were seen feeding. At Ste-Sophie, a greater percentage of gulls were observed feeding during the nesting and rearing stages than later in the season. The percentage of gulls feeding on garbage also varied throughout the daily periods at Ste-Sophie (*F*_2,26_ = 10.77, *p* < 0.001) with a greater percentage after 1500 (27% ± 6%) compared to morning (6% ± 2%) or mid-day (4% ± 2%). There was no difference between daily periods at the Terrebonne site (*F*_2,25_ = 1.50, *p* = 0.243).

### 3.2. Foraging Trips

We tracked 22 individuals during 41 foraging trips from the Deslauriers Island colony towards the Terrebonne (*n* = 28) and Ste-Sophie site (*n* = 13). This represented 18% of the 122 gulls tracked during the study and 10% of the total number of foraging trips (*n* = 418). At Terrebonne, seven trips (25%) resulted in a stopover that lasted 22 ± 7 min. This was significantly less frequent and of shorter duration than the 11 trips (85%) with a stopover at Ste-Sophie that lasted 63 ± 15 min (frequency: χ^2^ = 13.57, *p* < 0.001, duration: *t*_16_ = 2.54, *p* = 0.024). At Ste-Sophie, the three trips that took place during weekends all resulted in a stopover, whereas five out of the eight stopovers recorded during weekdays occurred before or after the working hours of the deterrence employee.

### 3.3. Gulls’ Mortality

At the Ste-Sophie landfill, a total of 180 gulls were culled over the 32-day trial period in 2010 for a mean of 5.6 per day. Juveniles began to be culled on June 24th, shortly after the first birds fledged from Deslauriers Island. They represented 15% of the total number of birds killed. At the Terrebonne landfill, 16 gulls were caught by the trained birds of prey during the 124 days of intensive observation in 2010 for an average of 0.10 dead gull per day. The program based on falconry thus resulted in 56 times less gulls being killed.

## 4. Discussion

The intensive integrated program that combined falconry, pyrotechnics and distress calls was successful in reducing the number of ring-billed gulls at the Terrebonne landfill. This was true when considering the long-term annual use of the site as well as the detailed observations conducted in 2010 at the Terrebonne and Ste-Sophie landfills. Although the two sites and their associated deterrence methods could not be directly compared, less gulls visited the Terrebonne landfill that was located 4.5 times closer to the colony and where 1.5 times more refuse was brought compared to the Ste-Sophie landfill. Based on these characteristics irrespective of the deterrence activities, we would have expected more gulls to use the Terrebonne site, but this was not the case.

Our study is unique because it spanned 20 years compared to other studies that lasted from a few weeks to a couple of years [16,17,18,24]. The major drawback of most deterrence methods is that birds become habituated to the scaring stimuli making these methods ineffective. We consider that the recurrent use of falconry combined with other complementary scaring techniques over several years contributed to discouraging ring-billed gulls that breed on Deslauriers Island from using the landfill.

Falconry involves a potentially lethal aspect that may reinforce its effect and impede habituation of gulls [17,24]. The amount of flying time by the birds of prey may appear limited (<2 h/day) but the birds are only flown when gulls are present. In addition, the impact of the birds of prey will persist for some time after the flight, which minimizes the time that the birds of prey need to be in the air.

The integrated program based on falconry succeeded in reducing the number of gulls to a level that was acceptable for both the site employees and the residents living in proximity to the landfill as indicated by a reduced number of complaints [25]. The number of gull-days decreased from over 1.1 million in 1995 to only 20,300 in 2014 while the number of gulls nesting at the nearby Deslauriers Island colony remained relatively high during the whole period. In fact, the local breeding population increased, remained stable and then declined during our study [20]. In addition, the amount of refuse brought to the site increased by nearly 50% between 1995 and 2014. The decline of the landfill use by gulls became more noticeable starting in 2005–2006 when the number of days with deterrence activities, the number of WCO and the number of hours per day devoted to deterrence were increased. We believe that the success of the integrated program based on falconry was explained by the limited opportunity (<1%) for the gulls to feed on refuse. The difficulty for gulls to obtain food at Terrebonne is also demonstrated by the smaller proportion of foraging trips that resulted in a stopover and the shorter duration of these stops compared to individuals that flew towards the Ste-Sophie landfill. Tracking individuals to evaluate the effectiveness of deterrence programs has never been used, and our results demonstrate the potential of this approach.

We showed that gulls use landfills to a greater extent during the rearing season when they have to travel back and forth to the colony to feed their juveniles. We observed more gulls during this period at the tipping area at Ste-Sophie where they spent more time feeding, especially after the working hours of the deterrence employee. We also recorded more gulls at the Terrebonne site during that stage but not at the tipping area. This was achieved by increasing the number of WCO and by maintaining the program during seven days a week even if refuse was not brought to the site on Sundays. This clearly shows the importance of adjusting the intensity of the deterrence programs to seasonal variation in bird use and to maintain the measures from dawn to dusk.

The cost of an integrated program based on falconry will obviously vary with the number of deterrence employees and the number of operation days per week but can amount to CAD$1,250 per week when gull abundance is low up to CAD$4,000 during the chicks’ rearing period. This includes the cost of maintaining the trained birds of prey, the specialized employees’ salary, the scaring material and vehicles. Although the use of falconry may appear more expensive than other methods, the results in reducing gull use at a site may warrant the expenses. This is especially true when the landfill is located near a gull colony, in urban or suburban settings or in the vicinity of an airport.

The use of birds of prey resulted in a much lower number of gulls killed compared to culling. In North America, gulls are protected under the Migratory Birds Convention Act and management measures like culling require a permit. Although the killing of gulls may not be an issue and may even be desirable to people living near a landfill, this may not be the case for a majority of citizens. Groups opposing to the killing of animals to control nuisance species are increasing and better organized with a greater impact on politicians [26,27]. Wildlife authorities may thus become more reluctant to issue culling permits, especially for species other than ring-billed gulls such as herring, great black-backed or glaucous gulls (*L. hyperboreus*) that were more abundant at the studied landfills in late fall and winter.

## 5. Conclusions

We consider that falconry is a more ethical method than culling to deter gulls from landfills because fewer birds are killed. A bird of prey catching a gull may also appear more natural than killing it by shooting. Nonetheless, some people may argue that non-lethal deterrence programs are less ethical because they do not alleviate the problem of gull abundance but just move it elsewhere. These people will advocate that killing of nuisance or overpopulating wildlife may not be necessarily unethical because it might have a direct impact on population dynamics. However, culling may not affect the number of birds to the same extent when used as a deterrence method [19] than when it is specifically designed to reduce population size. Moreover, large scale culls may not always have the desired effect of reducing problems associated with abundant gull species because other demographic parameters such as emigration may have an impact at the metapopulation level [28]. On the other hand, the decreased use of profitable feeding sites like landfills may affect population dynamics. For instance, Giroux, *et al.* [20] reported a lower chick survival in the Deslauriers colony following the implementation of the deterrence program based on falconry at the nearby Terrebonne landfill.

We have shown that the use of falconry as part of an integrated program is a useful deterrence method at landfills. It also gets better public perception and is generally more socially acceptable than lethal culling [16]. It can even be used by landfill managers as an advertising and educational tool. In any case, monitoring of the site in terms of bird use should be conducted before the beginning of a deterrence program and while it is performed to allow adjustments.
